# Prosocial Behavior and Friendship Quality as Moderators of the Association Between Anxious Withdrawal and Peer Experiences in Portuguese Young Adolescents

**DOI:** 10.3389/fpsyg.2018.02783

**Published:** 2019-01-11

**Authors:** Miguel Freitas, António J. Santos, Olívia Ribeiro, João R. Daniel, Kenneth H. Rubin

**Affiliations:** ^1^William James Center for Research, ISPA – Instituto Universitário, Lisbon, Portugal; ^2^Department of Human Development and Quantitative Methodology, University of Maryland, College Park, College Park, MD, United States

**Keywords:** anxious withdrawal, peer group, friendship, psychosocial adjustment, peer-valued attributes, early adolescence

## Abstract

Anxious withdrawal has been associated consistently with adverse peer experiences. However, research has also shown that there is significant heterogeneity among anxiously withdrawn youth. Further, extant research has focused primarily on negative peer experiences and outcomes; little is known about the more successful social experiences of anxiously withdrawn youth. We explored the possibility that the association between anxious withdrawal and group-level peer outcomes (exclusion, victimization, and popularity) might be moderated by peer-valued behaviors (prosocial behavior), friendship relational attributes, and sex, even after accounting for the effects of being involved in a reciprocal best friendship. Peer nominations of psychosocial functioning, and self-reports of best friendships and friendship quality were collected in a community sample of 684 Portuguese young adolescents. Regression analyses revealed that more anxious withdrawn adolescents showed worst group-level peer outcomes, but that: (a) prosocial behavior buffered the positive association between anxious-withdrawal and peer exclusion, particularly for boys; (b) higher friendship quality was associated with lower risk of peer victimization for more anxious-withdrawn girls, but with a higher risk for more anxious withdrawn boys; and (c) higher friendship conflict buffered the positive association between anxious withdrawal and peer exclusion for boys. Results are discussed in terms of the implications of peer-valued characteristics on the peer group experiences of anxiously withdrawn young adolescents.

## Introduction

Anxious withdrawal is a form of social withdrawal comprising the consistent display of solitary, socially reticent, and anxious behavior when encountering both familiar and unfamiliar peers who may be potential social partners (Gazelle, 2008; Rubin et al., 2009; Rubin and Barstead, 2017). Shy, anxious, and solitary behavior has been well established as a strong risk factor for a wide range of socio-emotional difficulties throughout development. Particularly, when compared to more sociable peers, anxious withdrawn children suffer more interpersonal adversity such as low popularity, peer exclusion and victimization (Coplan et al., 2001; Gazelle and Ladd, 2003; Markovic and Bowker, 2015), as well as more intrapersonal difficulties, such as loneliness and depression (Gazelle and Rudolph, 2004; Markovic and Bowker, 2017). Recent studies have shown that not all anxious withdrawn youths maintain their status over time (e.g., Gazelle and Shell, 2017), although it has been considered a stable phenomenon. Whilst some evidence increases in anxious withdrawal, others appear to decrease their behavior to normative levels (Booth-LaForce and Oxford, 2008; Oh et al., 2008).

Most studies of anxious withdrawal have focused on its association with adjustment problems and how, and whether, this association may be moderated by negative individual characteristics (Gazelle and Rudolph, 2004; Chen and Santo, 2015; Smith et al., 2017). For example, children and adolescents who display both anxious solitude and aggressive behaviors have been characterized as extremely prone to experience peer rejection, victimization, and exclusion (Gazelle, 2008; Bowker et al., 2012). However, few researchers have addressed the issue from an alternative standpoint, such as focusing on the positive attributes that anxiously withdrawn individuals display that may serve to reduce/buffer their vulnerability to maladaptation (Ladd et al., 2011; Markovic and Bowker, 2015). Moreover, most studies tend to focus on only one behavioral dimension of anxiously withdrawn individuals (withdrawn behavior, victimization, or prosocial behavior), rather than considering how different dimensions co-occur within them (Monahan and Booth-LaForce, 2016).

Of particular interest for this study is the display of prosocial behavior by anxious withdrawn youth. Prosocial behavior – intentional and voluntary behavior that benefits another, such as helping, sharing, and comforting (e.g., Eisenberg et al., 2006) – has been described as an important marker of peer acceptance and social competence (e.g., LaFontana and Cillessen, 2002; Sebanc, 2003). There is evidence that some anxiously withdrawn adolescents, despite not seeking social contact, are still able to display behaviors that are valued and appreciated by their peers (e.g., Asendorpf, 1994; Gazelle, 2008; Rubin et al., 2009; Monahan and Booth-LaForce, 2016). For example, Gazelle (2008) revealed that some anxious withdrawn children are considered friendly, nice and cooperative by their peers. This specific group of agreeable anxious solitary children experienced positive peer relations and low peer adversity. Thus, it is plausible that the display of prosocial behaviors by anxious withdrawn youth may act as a protective factor in peer group experiences.

Prior research on shy withdrawn youth’s poor relationships with peers is also limited because it has been focused on group-level outcomes, such as peer rejection (Rubin et al., 2015). Few studies have taken into account dyadic-level variables, such as friendship involvement (Markovic and Bowker, 2015) or, perhaps most importantly, friendship quality, when predicting the social adjustment of anxious withdrawn adolescents.

Shy anxious adolescents are as likely as their non-withdrawn counterparts to form and to maintain stable friendships (Schneider, 1999; Rubin et al., 2006). Anxious withdrawn youth who have a best friend evidence fewer internalizing and externalizing problems (Laursen et al., 2007; Markovic and Bowker, 2017), are less victimized (Ladd et al., 2011) and are perceived by peers as more socially accepted than those who lack best friends (Rubin et al., 2006). However, longitudinal studies have suggested that having a best friend might also be a risk factor for increasing shy-withdrawn behavior (e.g., Oh et al., 2008).

Notwithstanding, most previous studies are limited because they have not controlled for the effects of best friendship involvement (Markovic and Bowker, 2015) and, thus, it is still unclear whether anxiously withdrawn behavior is associated uniquely with group-level peer outcomes (e.g., peer exclusion and victimization, popularity) after accounting for the effects of having at least one best friend. The protective power associated with close friendships does not derive solely from having friends *per se*, but also from the quality of such relationships (Hartup, 1996). Evidence suggests that a high-quality relationship with a best friend, characterized by experiences of support, intimacy, self-disclosure, and acceptance (Parker and Asher, 1993), can be an important tool in promoting positive developmental outcomes, such as social adjustment, perspective-taking and social-cognitive skills (e.g., Steinberg, 2010; Rubin et al., 2015). Rich and supportive friendships have, thus, been posited as an important factor diminishing the negative consequences of group-level peer difficulties. Conversely, negative interactions within a best friendship may act as a risk factor for the development of maladaptive patterns of social behavior (Monahan and Booth-LaForce, 2016).

Some authors have suggested that the friendships of anxious withdrawn children are poorer in quality (e.g., Rubin et al., 2006), because they tend to befriend other shy, anxious children who share their behavioral style and, therefore, the same psychosocial problems (e.g., Haselager et al., 1998; Schneider, 1999; Rubin et al., 2006; Oh et al., 2008). Anxious withdrawn young adolescents also have more immature conceptions about friendship than their non-withdrawn counterparts, such that they unilaterally focus on their individual needs, rather than on intimacy and reciprocity (Schneider and Tessier, 2007). Moreover, some studies indicate that, for shy adolescents, supportive friendships may not be as beneficial as for non-withdrawn peers. For example, Barstead et al. (2017) have shown that more friendship support exacerbated the association between shyness and depression, and aggravated the difficulties faced by anxious withdrawn youth.

Together, such characteristics of anxious withdrawn youth’s close relationships may hinder their abilities to provide social support to their friends, and engage in intimate disclosure, a hallmark of friendships from early adolescence on (Rubin et al., 2015). Those relationships, rather than facilitating access to the peer group, appear to increase trajectories of anxious withdrawal (e.g., Oh et al., 2008) or psychological difficulties (Markovic and Bowker, 2017).

From early adolescence on, being accepted and popular becomes highly valued (LaFontana and Cillessen, 2010) and youth develop an increasing sensitivity to social behaviors that deviate from sociability, such as shyness (Rubin et al., 2009). It is therefore important to clarify the meaning and significance of social behaviors and relationships, such as anxious withdrawal and friendship, in a cultural context (Rubin et al., 2015) that is underrepresented in the literature. At present, researchers have yet to examine the significance of friendship for anxiously withdrawn Portuguese youth. Most studies of anxious withdrawn behavior have involved samples in North America (e.g., Rubin, 1993; Gazelle and Rudolph, 2004; Rubin et al., 2006), Asia (e.g., China – Chen et al., 2014, 2015), Australia (e.g., Gullone et al., 2006), Cuba (Valdivia et al., 2005), South America (e.g., Saldarriaga et al., 2012), and some countries in Europe (e.g., Italy, Finland and Netherlands – Cillessen et al., 1992; Casiglia et al., 1998; Sette et al., 2014; Ojanen et al., 2017). Few, if any, studies have been published on samples based in Portugal and, therefore, virtually nothing is known about the causes, correlates and consequences of withdrawn behavior among Portuguese youth. When compared with the European average, support to others, equality and solidarity are more salient values in Portugal (European Commission, 2012). Consistent with such cultural values of social relationships, competent behaviors such as helping, sharing and cooperating are considered important features of positive social interactions and may lead to different associations between social behaviors and social adjustment outcomes than previously found in other contexts (Rubin et al., 2015).

Literature also warrants that further clarification is needed regarding the relation between sex and both the correlates and outcomes of shyness during childhood and adolescence (e.g., Rubin and Barstead, 2014). It has been suggested that socially withdrawn boys tend to be at greater developmental risk than girls (Doey et al., 2014), particularly because shyness comprises a violation of the gender-expected behavior of dominance and assertiveness in boys. On the other hand, co-occurring prosocial and withdrawn behaviors might also be associated with distinct social benefits according to sex. For example, Gazelle (2008) reported that such pattern is related to higher exclusion and victimization for boys, but not for girls. Markovic and Bowker (2015), on the contrary, have found no sex differences in the positive association between anxious-withdrawal and overt victimization. A more careful review of available empirical evidence (see Rubin and Barstead, 2014) reveals inconclusive findings regarding sex differences in the prevalence, correlates, and consequences of anxious withdrawal.

Regarding friendship dimensions, the prevalence of mutual best friendships does not appear to differ between boys and girls (Rubin et al., 2006; Ladd et al., 2011), but previous findings have shown that girls favor more intimate relationships, whereas boys favor belonging to a wider group of peers (see Rose and Rudolph, 2006, for a review). Moreover, girls describe their friendships as being of higher quality than do boys (e.g., Parker and Asher, 1993). In light of this mixed evidence, sex will be explored as a possible moderator.

### Current Research

The present study builds on the limitations of past research by examining: (a) the concurrent associations between anxious withdrawal and experiences within the peer group (exclusion, victimization, and popularity), while taking into account best friendship involvement; and (b) how these associations are moderated by peer-valued behaviors (prosocial behavior), the quality of the best friendship and sex.

Based on the extant, relevant literature presented above, we predict a positive association between anxious withdrawal and peer group adversity (i.e., exclusion and victimization), and a negative association between anxious withdrawal and peer group success (i.e., popularity). We also predict that these associations involving anxious withdrawal are buffered by higher levels of prosocial behaviors and friendship quality. Due to the inconsistent findings in the literature, no specific predictions are formulated as to possible sex differences.

## Materials and Methods

### Participants

Participants were drawn from a longitudinal normative sample from two public schools in the Lisbon Metropolitan area, from middle class neighborhoods (recruitment rate was around 85% of the overall school population). The sample for this study comprised 684 students (374 girls) from 28 seventh- and eighth-grade classes, whose ages ranged from 11 to 13 years old (*M* = 12.41, *SD* = 0.60). Mean class size was 24.26 students (*SD* = 2.23).

### Measures

#### Peer Nominations

Participants completed the Portuguese version of the Extended Class Play (ECP; Burgess et al., 2006; Correia et al., 2014), which assesses peers’ evaluations of the participants’ social functioning and reputation. We instructed adolescents to pretend to be the directors of an imaginary class play and to nominate one boy and one girl from among their participating classmates for each of 37 positive and negative roles. Consistent with past research (e.g., Zeller et al., 2003; Rubin et al., 2006), we only considered same-sex nominations to eliminate possible sex-stereotyping. We summed and standardized within sex and within class the nominations received by each participant, for each role, to adjust for class size differences (i.e., for differences in the number of potential nominators) (Cillessen, 2009). Finally, we calculated the following mean composites by averaging the corresponding standardized individual item scores: (1) Anxious withdrawal (three items; “Very shy,” “Doesn’t talk much or talks quietly,” “Rarely starts up conversations”; Cronbach’s α = 0.88); (2) Peer exclusion (three items; “Trouble making friends,” “Often left out,”” Can’t get others to listen”; α = 0.83); (3) Peer victimization (three items; “Mean things said to,” “Gets picked on,” “Hit or kicked by others”; α = 0.89); (4) Prosocial behavior (four items; “Helps others,” “Plays fair,” “Trustworthy,” “Has good ideas”; α = 0.76); and (5) Popularity/sociability (four items; “Everyone likes,” “Makes new friends easily,” “Has many friends,” “Likes to play with others”; α = 0.77).

The ECP has been used extensively in research on children’s behavioral and reputational characteristics and is highly reliable, because it is based on nominations provided by children’s peer group (see Cillessen, 2009; Rubin et al., 2015 for reviews). The ECP has been validated in different cultural settings (e.g., Rubin et al., 2006; Menzer et al., 2010; Bowker and Raja, 2011), including Portugal (Correia et al., 2014).

#### Best Friendship Involvement

We asked participants to nominate their class “very best friend” and “second best friend” (Bukowski et al., 1994). Consistent with past research (Parker and Asher, 1993; Rubin et al., 2006), we restricted nominations to two same-sex participating classmates, because it has been suggested that, relative to other good friendships, the closest (best) friendship relationships have greater impact on emotional and social development (e.g., Urberg et al., 1998). In line with previous studies (e.g., Parker and Asher, 1993), 62% of participants were involved in at least one reciprocal best friendship. We considered a reciprocal best friendship existed whenever both adolescents were each other’s first or second-best friend choice. We coded this categorical variable as 0 for no-reciprocal best friend and 1- at least one reciprocal best friend, in line with the procedure used in previous studies (e.g., Markovic and Bowker, 2015).

#### Friendship Positive Quality

We asked all participants to report on the quality of the relationship they had with the classmate they considered to be their best friend (identified via friendship nominations, independently of whether or not that choice was reciprocal) using the Friendship Quality Questionnaire (FQQ; Parker and Asher, 1993). The FQQ is a 40 item self-report questionnaire, which includes five positive dimensions: companionship and recreation (five items; e.g., “My friend and I do fun things together a lot”), validation and caring (seven items; e.g., “My friend and I make each other feel important and special”), help and guidance (nine items; e.g., “My friend and I help each other with schoolwork a lot”), intimate disclosure (five items; e.g., “My friend and I are always telling each other about our problems”), and conflict resolution (three items; e.g., “If my friend and I get mad at each other, we always talk about how to get over it”). This structure has been previously validated and confirmed in a Portuguese sample (Freitas et al., 2013).

Participants rated how true each FQQ item was for their best friend relationship on a 5-point Likert-type scale (1 = not true and 5 = very true). Higher scores indicated greater perceived friendship quality. Because the positive FQQ subscales were highly correlated (range: *r* = 0.56 to 0.79), as in previous studies (Fordham and Stevenson-Hinde, 1999; Rubin et al., 2006), we computed a total friendship positive quality score for each participant (α = 0.95).

#### Friendship Conflict and Betrayal

We used the conflict and betrayal dimension of FQQ to measure the negative features of best friendships’ quality (seven items; e.g., “My friend and I get mad at each other a lot,” “My friend sometimes says mean things about me to other kids”). Higher scores indicated greater perceived conflict and betrayal within the relationship with best friend (α = 0.87).

### Procedure

This study was approved by ISPA-Instituto Universitário’s Ethical Committee. We obtained authorization to conduct the study from the schools boards’, as well as written informed consent and/or assent from all participants’ caregivers and participating youth.

Personal data collection and processing followed the Declaration of Helsinki, APA guidelines and the European General Data Protection Regulation, ensuring the privacy and confidentiality of participants’ information.

During the spring semesters, research assistants administered a battery of questionnaires, in group format, within the adolescents’ classrooms. Each session lasted approximately 90 min. Non-participating adolescents remained in the classroom during data collection sessions, completing tasks assigned by their teacher.

#### Data Analytic Plan

The primary goal of the present study was to test the association between anxious withdrawal and peer group outcomes, as well as the potential moderating effects of prosocial behavior, best friendship quality, best friendship conflict and sex. In order to accomplish this goal, we ran a separate multiple linear regression model for each of the dependent variables^1^: peer exclusion, peer victimization and popularity/sociability. The following three-way interactions were included in the models: (1) anxious withdrawal × prosocial behavior × sex, (2) anxious withdrawal × friendship positive quality × sex, and (3) anxious withdrawal × conflict/betrayal × sex. We also included corresponding lower order interactions (seven two-way interactions) and main effects. We included as well the main effect of best friendship involvement in the model as a control variable. Prior to modeling, we standardized friendship positive quality and conflict within classes. All main effects (anxious withdrawal, prosocial behavior, best friendship involvement, quality and conflict, as well as sex) and interaction effects are displayed in Table 2. As such, and to ease presentation, only results pertaining to interaction effects are described below.

Models were fitted using stats package in R version 3.5.0 (R Core Team, 2018). To explore interaction effects, we conducted simple slope analyses using jtools package in R (Long, 2018). We provided graphical presentations below to detail these effects.

## Results

Table 1 shows descriptive statistics and correlations for all the studied variables. Briefly, zero-order correlations values showed that associations between anxious withdrawal and peer outcomes (without controlling for other variables) followed predicted relations patterns. Higher anxious withdrawal scores were positively correlated with negative peer outcomes (exclusion and victimization; *r* = 0.51 and *r* = 0.27, respectively) and negatively correlated with popularity/sociability (*r* = -0.16). This correlation pattern is indicative of a more general trend showing that variables with a positive valence (popularity/sociability, prosocial behavior, friendship involvement) were positively correlated with each other, and negatively correlated with negative valence variables (exclusion, victimization, anxious withdrawal, and conflict/betrayal).

**Table 1:** Means, SD, and zero-order Pearson correlations between study variables.

		2	3	4	5	6	7	8	9	*M*	*SD*	*N*
	**Dependent variables**											
1	Peer exclusion	0.65	-0.25	0.51	-0.17	-0.20	0.00	-0.13	0.00	0.00	0.79	670
2	Peer victimization		-0.18	0.27	-0.17	-0.16	0.03	-0.15	0.00	0.00	0.83	672
3	Popularity/sociability			-0.16	0.45	0.16	-0.10	0.17	0.00	0.00	0.71	672
	**Predictors**											
4	Anxious withdrawal				0.02	-0.15	-0.04	0.02	0.00	0.00	0.80	660
5	Prosocial behavior					0.17	-0.20	0.26	0.00	0.00	0.70	672
6	Friendship positive quality						-0.12	0.20	-0.23	3.68	0.84	561
7	Conflict/betrayal							-0.08	0.11	1.77	0.78	580
8	Friendship involvement								-0.11	0.62		609
9	Sex (boys)									0.45		684


*Friendship involvement (no = 0 and yes = 1); sex (girls = 0 and boys = 1); mean values of the categorical values equal the proportion of category = 1.*

### Peer Exclusion

**FIGURE 1:**
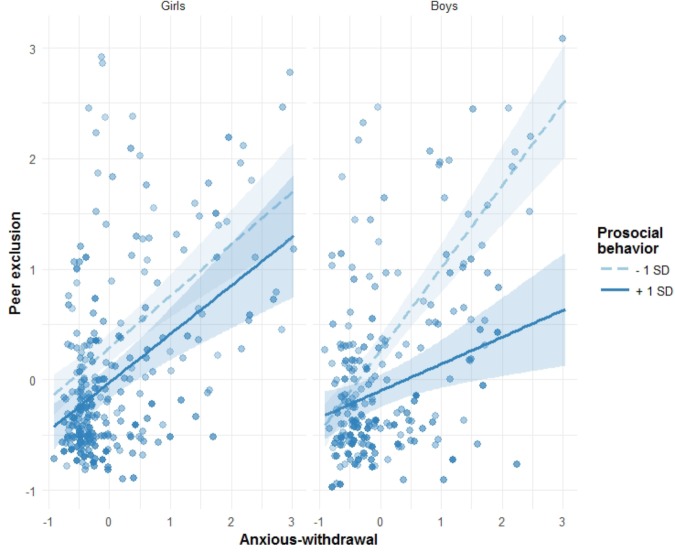
Prosocial behavior as a moderator of the association between anxious withdrawal and peer exclusion, separately for girls and boys. 95% confidence intervals are displayed around simple slope lines (see Table 3 for more details). Points in the graph are shaded according to prosocial behavior scores.

When predicting peer exclusion, the significant main effects of anxious withdrawal and prosocial behavior were qualified by three-way interactions (Table 2): (1) anxious withdrawal × sex × prosocial behavior (*β*_14_ = -0.34) and (2) anxious withdrawal × sex × conflict/betrayal (β_16_ = -0.23). For the first interaction (see Table 3 and Figure 1), simple slope analysis indicated that the association between anxious withdrawal and peer exclusion, although differing in magnitude, was always positive, independently of sex and prosocial behavior levels (i.e., as shown in Table 3, all simple slope estimates are positive; Figure 1 shows that all lines have a positive slope). In both graphs of Figure 1, prosocial behavior slopes showed that higher prosocial levels (1 SD slopes in Figure 1; SS2 and SS4 in Table 3) associated with lower levels of peer exclusion in both boys and girls. In other words, anxious withdrawn adolescents who evidenced more prosocial behavior were less excluded than similarly anxious withdrawn counterparts with lower levels of prosocial behavior. This buffering effect of prosocial behavior was more evident for highly anxious withdrawn boys.

**Table 2:** Regression models of peer exclusion, victimization and popularity/sociability.

		Exclusion (*R*^2^ = 0.36)				Victimization (*R*^2^ = 0.17)				Popularity/Sociability (*R*^2^ = 0.26)			
				
		Estimate	*SE*	*t*	*p*	Estimate	*SE*	*t*	*p*	Estimate	*SE*	*t*	*p*
β*0*	Intercept	0.15	0.06	2.50	0.013^*^	0.20	0.07	2.82	0.005^**^	-0.03	0.06	-0.51	0.613
	**Main effects**												
* β1*	Friendship involvement	-0.14	0.06	-2.21	0.027^*^	-0.22	0.08	-2.92	0.004^**^	0.05	0.06	0.82	0.414
β*2*	Sex (boys)	-0.05	0.06	-0.80	0.422	-0.03	0.07	-0.36	0.716	0.08	0.06	1.36	0.176
β*3*	Anxious withdrawal	0.45	0.05	9.01	<0.001^***^	0.18	0.06	2.88	0.004^**^	-0.27	0.05	-5.43	<0.001^***^
β*4*	Prosocial behavior	-0.22	0.06	-3.88	<0.001^***^	-0.24	0.07	-3.37	<0.001^***^	0.36	0.06	6.50	<0.001^***^
β*5*	Friendship positive quality	-0.11	0.05	-2.38	0.018^*^	-0.10	0.06	-1.75	0.081	0.09	0.04	1.95	0.052 .
β*6*	Conflict/betrayal	-0.03	0.04	-0.61	0.541	-0.04	0.05	-0.81	0.421	0.02	0.04	0.53	0.596
	**Two-way interaction effects**												
β*7*	Anxious withdrawal × prosocial behavior	-0.02	0.09	-0.25	0.802	0.00	0.11	0.04	0.972	-0.14	0.08	-1.65	0.100
β*8*	Anxious withdrawal × friendship positive quality	-0.08	0.06	-1.45	0.147	-0.09	0.07	-1.25	0.212	-0.03	0.06	-0.58	0.563
β*9*	Anxious withdrawal × conflict/betrayal	0.06	0.05	1.15	0.253	-0.05	0.07	-0.83	0.406	-0.08	0.05	-1.57	0.117
β*10*	Anxious withdrawal × sex	0.07	0.08	0.87	0.385	0.18	0.09	1.97	0.050^*^	0.11	0.07	1.49	0.138
β*11*	Sex × prosocial behavior	-0.04	0.08	-0.45	0.656	-0.01	0.10	-0.06	0.954	0.07	0.08	0.88	0.381
β*12*	Sex × friendship positive quality	0.07	0.06	1.07	0.288	0.07	0.08	0.97	0.331	-0.08	0.06	-1.27	0.205
β*13*	Sex × conflict/betrayal	0.00	0.06	0.05	0.957	0.06	0.08	0.79	0.433	-0.09	0.06	-1.53	0.127
	**Three-way interaction effects**												
β*14*	Anxious withdrawal × sex × prosocial behavior	-0.34	0.12	-2.75	0.006^**^	-0.27	0.15	-1.84	0.067	-0.03	0.12	-0.22	0.823
β*15*	Anxious withdrawal × sex × friendship positive quality	0.11	0.08	1.46	0.145	0.19	0.09	2.03	0.043^*^	0.02	0.07	0.32	0.749
β*16*	Anxious withdrawal × sex × conflict/betrayal	-0.23	0.07	-3.07	0.002^**^	-0.10	0.09	-1.08	0.281	0.10	0.07	1.34	0.183


*Estimates involving continuous predictors refer to how many units dependent variables are predicted to change, per unit increase in the predictor variable; estimates involving categorical predictors refer to how many units dependent variables are predicted to differ between categories; friendship involvement (no = 0 and yes = 1); sex (girls = 0 and boys = 1). ^∗^*p* < 0.05, ^∗∗^*p* < 0.01, and ^∗∗∗^*p* < 0.001.*

**Table 3:** Simple slope analysis of the anxious withdrawal × sex × prosocial behavior interaction effect on peer exclusion.

	Prosocial behavior	Estimate	*SE*	*t*	*p*
**Girls**					
SS1	-1 SD	0.47	0.07	6.28	<0.001^∗∗∗^
SS2	+1 SD	0.44	0.08	5.35	<0.001^∗∗∗^
**Boys**					
SS3	-1 SD	0.77	0.08	9.04	<0.001^∗∗∗^
SS4	+1 SD	0.27	0.08	3.49	<0.001^∗∗∗^


*SS, simple slope; estimates refer to how many units peer exclusion is predicted to change, per unit increase in anxious withdrawal, according to prosocial behavior levels and sex.*

**FIGURE 2:**
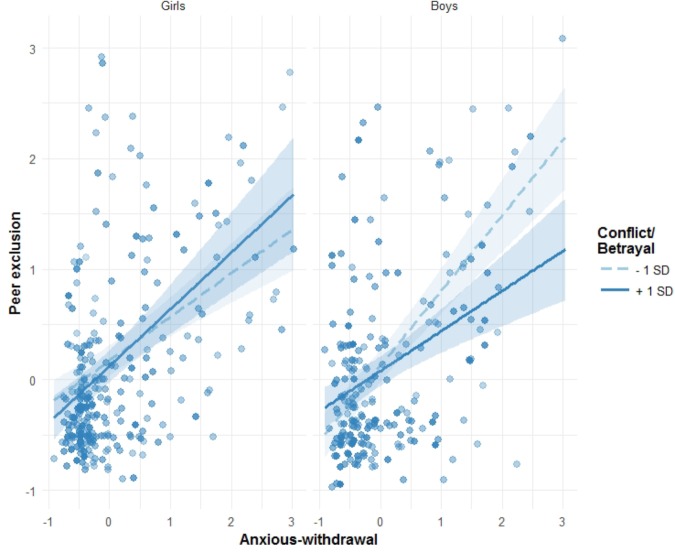
Friendship conflict and betrayal as a moderator of the association between anxious withdrawal and peer exclusion, separately for girls and boys. 95% confidence intervals are displayed around simple slope lines (see Table 4 for more details). Points in the graph are shaded according to conflict/betrayal scores.

For the second three-way interaction referred (anxious withdrawal × sex × conflict/betrayal) the simple slope analysis (see Table 4 and Figure 2) indicated that the association between anxious withdrawal and peer exclusion was also moderated by conflict/betrayal, but in this case only for boys. Again, the association between anxious withdrawal and peer exclusion, although differing in magnitude, was always positive, independently of sex and conflict/betrayal levels (i.e., Table 4 shows that all simple slope estimates are positive; in Figure 2, all lines have a positive slope). Nevertheless, as we referred, the moderating effect of conflict/betrayal on the association between anxious withdrawal and peer exclusion differed for boys and girls. Figure 2 shows that, in girls, regression lines for the different levels of conflict/betrayal ran very close to each other (see also SS2 and SS2; Table 4); but for boys, these lines diverge (see also SS3 and SS4; Table 4). Conflict/betrayal levels thus moderated the association between peer exclusion and anxious withdrawal for boys, but not so for girls. In other words, higher conflict/betrayal in anxious withdrawn boys was associated with lower peer exclusion (boys 1 SD line in Figure 2 appears below -1 SD line), while for anxious withdrawn girls different conflict/betrayal levels associated with more similar levels of peer exclusion.

**Table 4:** Simple slope analysis of the anxious withdrawal × sex × conflict/betrayal interaction effect on peer exclusion.

	Conflict/betrayal	Estimate	*SE*	*t*	*p*
**Girls**					
SS1	-1 SD	0.40	0.06	6.84	<0.001^∗∗∗^
SS2	+1 SD	0.51	0.08	6.44	<0.001^∗∗∗^
**Boys**					
SS3	-1 SD	0.67	0.07	9.20	<0.001^∗∗∗^
SS4	+1 SD	0.37	0.07	5.23	<0.001^∗∗∗^


*SS, simple slope; estimates refer to how many units peer exclusion is predicted to change, per unit increase in anxious withdrawal, according to conflict/betrayal levels and sex.*

### Peer Victimization

**FIGURE 3:**
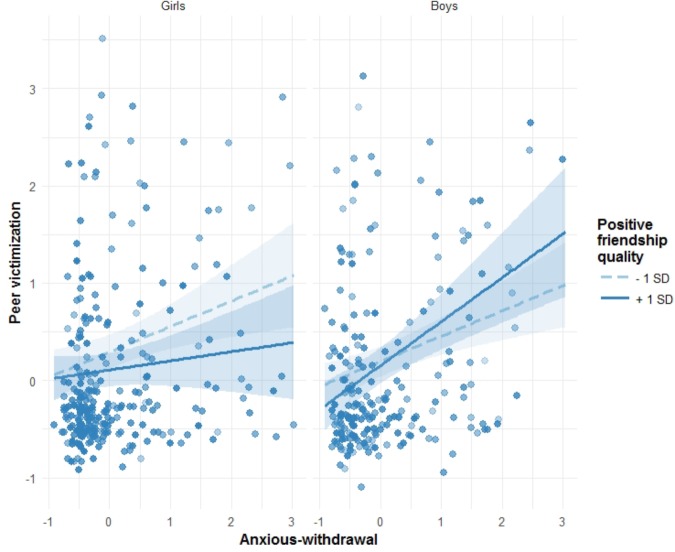
Friendship quality as a moderator of the association between anxious withdrawal and peer victimization, separately for girls and boys. 95% confidence intervals are displayed around simple slope lines (see Table 5 for more details). Points in the graph are shaded according to friendship positive quality scores.

The regression model predicting peer victimization revealed again, as for peer exclusion, that the significant main effect of anxious withdrawal was qualified by a three-way interaction (Table 2): anxious withdrawal × sex × friendship positive quality (*β*_15_ = 0.19). Simple slope analysis (see Table 5 and Figure 3) indicated that the association between anxious withdrawal and peer victimization, although different in magnitude, was always positive, independently of sex and friendship positive quality levels (i.e., in Table 5, all simple slope estimates are positive; in Figure 3, all lines have a positive slope). In sum, more anxious withdrawn adolescents were more victimized by peers. Figure 3 also shows that the moderator effect of friendship positive quality was present in both boys and girls. But while higher levels of self-reported friendship positive quality in girls buffered the association between anxious withdrawal and peer victimization (girls 1 SD line appears below -1 SD line), in boys the effect was reversed (boys 1 SD line above -1 SD line). That is, higher levels of friendship positive quality in boys augmented the deleterious association between anxious withdrawal and peer victimization.

**Table 5:** Simple slope analysis of the anxious withdrawal × sex × friendship positive quality interaction effect on peer victimization.

	Friendship positive quality	Estimate	*SE*	*t*	*p*
**Girls**					
SS1	-1 SD	0.25	0.08	2.97	0.003^∗∗^
SS2	+1 SD	0.10	0.09	1.16	0.245
**Boys**					
SS3	-1 SD	0.27	0.07	3.88	<0.001^∗∗∗^
SS4	+1 SD	0.44	0.10	4.44	<0.001^∗∗∗^


*SS, simple slope; estimates refer to how many units peer victimization is predicted to change, per unit increase in anxious withdrawal, according to friendship positive quality levels and sex.*

There was also a positive, significant two-way interaction between anxious withdrawal and sex (*β*_10_ = 0.18; Table 2), indicating that the association between anxious withdrawal and peer victimization was stronger for boys than girls.

### Popularity/Sociability

The popularity/sociability regression (Table 2) model showed significant main effects for anxious withdrawal (*β*_3_ = -0.27) and prosocial behavior (*β*_4_ = 0.36). That is, more prosocial youth scored higher on popularity/sociability, while more anxiously withdrawn youth scored lower on popularity/sociability. None of these main effects was qualified by an interaction effect, and no other significant interaction effects were found.

## Discussion

The developmental significance of social interactions and relationships, specifically during adolescence, has been well-established (see Rubin et al., 2015 for a review). Particularly noteworthy are findings that some social-behavioral characteristics have been consistently associated with poor peer outcomes. For example, researchers have found that anxious withdrawn youth are often rejected, excluded, and victimized by their peers (e.g., Rubin et al., 2006; Oh et al., 2008; Markovic and Bowker, 2015). Yet, it is also the case that not all anxiously withdrawn youth share identical developmental trajectories (e.g., Gazelle, 2008). Our results show that there are specific behavioral and relationship factors that protect anxiously withdrawn youth from negative social outcomes (see also Gazelle, 2008; Ladd et al., 2011; Markovic and Bowker, 2015).

Globally, the main goals of this study were to test: (a) the concurrent associations between anxious withdrawal and both negative and positive peer group outcomes (peer exclusion, peer victimization and popularity/sociability), while controlling for best friendship involvement and (b) whether these associations were moderated by peer-valued behaviors (prosocial behavior), the quality of the best friendship and sex.

Our results contribute to a growing body of research carried out in distinct cultural contexts (e.g., Gullone et al., 2006; Rubin et al., 2006; Sette et al., 2014; Chen et al., 2015; Ojanen et al., 2017), by showing that Portuguese anxiously withdrawn adolescents experience greater difficulties and less success in their contact with the peer group. Consistent with prior research (e.g., Rubin et al., 2006), we found that anxious withdrawal significantly predicted higher levels of peer exclusion and victimization, as well as lower popularity/sociability.

This study is one of the few to demonstrate that the deleterious effects of anxious withdrawal go beyond the mere existence of a reciprocal best friendship. Past research has suggested that adolescents who refrain from interacting (or who are viewed by peers as overtly shy, anxious and reticent) display behavioral patterns that are inconsistent with peers’ expectations (Rubin et al., 2006; Asendorpf, 2010). Consequently, these youth are considered to be less competent and suffer from low social preference and status. By removing themselves from the school social scene, they are regarded as fragile, vulnerable, lonely and helpless, becoming easy targets for exclusion and victimization (Gazelle and Ladd, 2003; Rubin et al., 2006; Gazelle, 2008; Markovic and Bowker, 2015).

Another important finding of the present study is that it showed a beneficial association between prosocial behavior and peer outcomes. More prosocial adolescents were less likely to be excluded and victimized by classmates and were considered by them to be more popular. These results are consistent with previous studies that also report greater support in peer relationships (Sebanc, 2003), greater acceptance and popularity (e.g., LaFontana and Cillessen, 2002; Hawley, 2003; Markovic and Bowker, 2015) and lower victimization (Monahan and Booth-LaForce, 2016) for children and youth who exhibit socially appropriate and competent behaviors such as helping, sharing, caring, and cooperating.

More relevant, and as predicted, we found prosocial behavior to play a protective role for anxiously withdrawn adolescents. Those who displayed higher levels of prosocial behavior experienced lower peer exclusion than their less prosocial counterparts. This effect was especially strong for boys. This finding is consistent with the diathesis-stress model of anxious withdrawal proposed by Gazelle and Ladd (2003) that suggests an association between decreasing peer adversity, experienced in the form of exclusion, and increasing prosocial behavior (Gazelle and Rudolph, 2004; Gazelle, 2008). Drawing on the motivational model of social withdrawal, shy, withdrawn adolescents are conceptualized as experiencing a desire to interact with their peers that is counteracted by a simultaneous motivation to avoid social contact (Asendorpf, 1990). Thus, just as some aggressive children that display prosocial strategies obtain a more favorable position within the peer group (Hawley, 2003), it may be the case that some anxiously withdrawn individuals display helping, caring, and sharing behaviors toward their peers. Previous research, however, has suggested that prosocial behavior might be interpreted as immature, attention-seeking and unskillful, especially in the case of anxiously withdrawn boys, whom accumulate two distinct behaviors which are gender atypical and inconsistent with social expectations (Gazelle, 2008; LaFontana and Cillessen, 2010). For that reason, prosocial behaviors have been reported to augment the risk of suffering peer mistreatment for anxiously withdrawn children (Markovic and Bowker, 2015), particularly for boys (Gazelle, 2008). Notwithstanding, our findings suggest that being friendly, cooperative and trustworthy, even for boys, may actually be attributes that peers value and perceive as desirable, despite their timidity. Such interactive styles, which may serve as a gateway into forming dyadic relationships or into larger social groups (Gazelle, 2008), might divert anxiously withdrawn youth (particularly boys) from pathways with negative outcomes (Coplan et al., 2006; Rubin et al., 2009). This prosocial dimension might be particularly relevant in a cultural context where support to others, equality and solidarity are salient values, like Portugal (European Commission, 2012).

Friendships are posited by peer-relations theory to have a crucial role in protecting children and, more relevantly, adolescents from negative social experiences (e.g., Parker and Asher, 1993; Hartup, 1996; Rubin et al., 2015). Our results indicate that being involved in a reciprocal best friendship is predictive of lower peer adversity. In the case of youth who refrain from interacting with peers, researchers have been strongly urged to carefully explore the dyadic-level of social relationships (Rubin et al., 2015) in predicting anxiously withdrawn adolescents’ social adjustment. Given that the protective power associated with close friendships might not derive solely from having friends *per se*, but also from the quality of such relationships (Hartup, 1996), the current study tested whether the quality of best friendship served a buffering role against the negative effects of anxious withdrawal on peer outcomes. Our analyses showed that positive and negative features of adolescents’ best friendships revealed sex differences in predicting peer experiences of anxiously withdrawn youth. Experiencing a high-quality best friendship had a buffering effect of withdrawn behavior on peer exclusion, exclusively for girls. Conversely, for anxiously withdrawn boys, higher friendship positive quality emerged as a risk factor for peer victimization. Moreover, conflict and betrayal with a best friend also revealed mixed findings: for anxiously withdrawn girls, different levels of this feature did not predict major differences in peer exclusion; for anxiously withdrawn boys, surprisingly, high conflict within a best friendship appeared to buffer the risk of being excluded by the peer group.

Results regarding boys were unexpected and may seem counterintuitive, but they are in line with existing evidence connecting high quality friendships to maladaptive patterns of behavior (e.g., Schmidt and Bagwell, 2007; Dishion and Tipsord, 2011; Barstead et al., 2017; Noret et al., 2018). Anxiously withdrawn youth have been described as forming poorer quality friendships with other shy, anxious social partners who share the same behavioral style and, thus, the same psychosocial difficulties (e.g., Haselager et al., 1998; Schneider, 1999; Rubin et al., 2006; Oh et al., 2008). Such characteristics of anxiously withdrawn youth’s friendships may compromise their abilities to provide social support to their friends, and engage in intimate disclosure, a hallmark of friendships from early adolescence on (Rubin et al., 2015). For example, anxiously withdrawn youth tend to consistently engage in co-rumination, i.e., the discussion of problems and hassles within a dyad that is not oriented toward a solution (Rose, 2002). This maladaptive style, which may be predicted by social anxiety (Jose et al., 2012), has also been linked to increased perception of friendship quality. To that end, shy withdrawn boys may not derive from their best friendships any protection or instrumental and social support they need in order to effectively overcome victimization by peers. In fact, our findings suggest that high friendship quality increased their risk for suffering peer mistreatment. Since boys typically function in large social networks (see Rose and Rudolph, 2006 for a review), perhaps the fact that anxious withdrawn boys remove themselves from the peer group at-large, to engage in more intimate and exclusive dyadic relationships (i.e., smaller social networks) might be once again perceived by peers as gender atypical and inconsistent with social expectations. This may, in turn, increase their salience as vulnerable and easy targets for abuse.

On the other hand, it has been suggested that, when compared to boys, who would be socialized to be more assertive and controlling, girls should adhere to a more caring and nurturing stereotype (see Rubin and Barstead, 2014, for a review). In that sense, shy, anxious withdrawn behavior might be more socially tolerable in girls and, as such, less adversely responded to by significant others (e.g., parents and peers) (Coplan et al., 2001; Doey et al., 2014). This process is thought to pose a greater risk for males than females of developing intra- and interpersonal difficulties. In fact, there is some evidence that socially withdrawn girls, but not boys, described themselves as being as socially skilled as their average peers (Rubin et al., 1993). For girls, then, whose social networks have been described as more exclusive and intimate, the importance of dyadic relationships (Rose and Rudolph, 2006) might find support in our results. Close, interpersonal bonds and, particularly, the positive interactions within those relationships may be, for anxiously withdrawn girls, powerful protective factors against peer mistreatment (Hartup, 1996; Boivin et al., 2001; Rubin et al., 2006). Literature indicates that rich, high quality friendships provide a safe context in which youth may experience support, intimacy, reciprocity, self-disclosure and validation, allowing them to elaborate on and learn from such experiences (e.g., Parker and Asher, 1993; Steinberg, 2010; Rubin et al., 2015). Therefore, drawing on past evidence that adolescents with high quality friendships deal with peer pressure and resolve conflicts more competently, our findings suggest that anxiously withdrawn girls also benefit from these friendship provisions and perhaps develop, to some extent, the ability to express disagreement and constructively discuss with peers (e.g., Cuadros and Berger, 2016).

Taken together, the current findings suggest that, when considering the effects of anxious withdrawal on psychosocial adjustment, not only different aspects of friendship quality may uniquely influence this association (above and beyond having a reciprocal best friend), but also that sex is a critical variable. In fact, distinct mechanisms may be in place for boys and girls in the links between anxious withdrawal and peer adversity. This possible explanation is merely speculative and the relationship between gender and anxious withdrawal must be better clarified (Rubin and Barstead, 2014). Therefore, future studies should seek to replicate these findings, in order to elucidate the dyadic mechanisms which may play a role in this process.

Our models also indicate that conflict and betrayal with a best friend moderated differently for girls and boys the link between anxious withdrawal and peer outcomes. For anxiously withdrawn girls, higher levels of this negative feature were not as strongly associated with peer exclusion as for boys. Boys who reported high perceived conflict in their best friendship were somewhat buffered against the effects of anxious withdrawal on peer exclusion. This finding is in line with previous literature showing that conflict may be normative and does not necessarily have detrimental effects on socioemotional adjustment (e.g., Laursen and Pursell, 2009). In fact, some researchers have suggested that conflict may be a socialization opportunity for adolescents to develop skills in negotiating, perspective-taking and coping with intra- and interindividual stress (e.g., Adams and Laursen, 2007). There is also some evidence that boys might be less affected by interpersonal conflict with peers than girls are (e.g., Ehrlich et al., 2012). Particularly for anxiously withdrawn boys, frequent interactions with best friends characterized by fighting and arguing may be perceived by peers as expressions of agency, a dimension that is most common in male friendships (Hall, 2011). Therefore, this attitude, which contrasts with the perspective that anxiously withdrawn youth are shy, timid, passive and submissive (e.g., Rubin et al., 2009), may be interpreted by peers as a sign of assertiveness and, to some extent, social competence.

Finally, our models do not intend to suggest that the variables examined are the only influences that peer experiences are subjected to. Future studies might also explore other aspects such as scholastic success or teacher-youth relationship (e.g., Arbeau et al., 2010), sense of humor, physical attractiveness and athletic ability (e.g., Markovic and Bowker, 2015), size of friendship network, or the identity and characteristics of the friends (Ladd et al., 2011). Notwithstanding, the moderation effects that were reported are not insignificant – in fact, each one holds the potential for future research or intervention that may contribute to a better understanding and a restriction of the impact of anxious withdrawal in adolescents’ social development.

Another important contribution of the present study was that multiple aspects of behavior were simultaneously considered when exploring adolescents’ psychosocial adjustment. For example, by revealing that some shy-withdrawn youth also behave, to some extent, prosocially toward peers (e.g., Gazelle, 2008; Monahan and Booth-LaForce, 2016), our findings enlighten the complexity of the behavioral patterns of anxiously withdrawn youth (e.g., Rubin et al., 2009).

One limitation of our study was the cross-sectional design of this study, which makes it impossible to draw firm conclusions in terms of the causal mechanisms that may underlie the reported associations. For that reason, findings are to be interpreted with caution. Further studies are warranted to replicate these findings, particularly with longitudinal designs in order to more fully capture and explicate how such factors impact peer experiences, psychosocial adjustment, and developmental trajectories of anxious withdrawal.

In addition, we used a generic measure of peer victimization, which did not disentangle different forms of victimization (e.g., overt and relational). Future studies should explore whether prosocial behavior and quality of best friendship play different roles for anxiously withdrawn boys and girls, in protecting or exacerbating the risk for distinct types of victimization.

In conclusion, the results of this investigation add to the pool of evidence pertaining to the difficulties that anxious withdrawn adolescents suffer in their daily experiences with peers at school. By controlling the involvement in best friendships, our findings suggest that the reported effects are specific to anxious withdrawal, above and beyond the presence or absence of reciprocal friendships. Our study contributes to the existing research by exploring some positive interactive and relationship attributes of anxiously withdrawn youth that may facilitate their integration in the peer group.

## Author Contributions

AS, KR, and MF designed the studies. MF and OR collected the data. MF and JD analyzed the data. AS, MF, OR, and JD interpreted the data and drafted the manuscript. AS, KR, OR, and JD critically revised the manuscript. All authors approved final version to be published.

## Conflict of Interest Statement

The authors declare that the research was conducted in the absence of any commercial or financial relationships that could be construed as a potential conflict of interest.

## Footnotes

1 Despite the nested structure of the data (children within classes), multilevel null models show that class level variability is ∼0 for the three dependent variables (making the use of multilevel procedures unnecessary).
